# Right Ventricular Septal Versus Apical Pacing: Long-Term Incidence of Heart Failure and Survival

**DOI:** 10.3390/jcdd9120444

**Published:** 2022-12-09

**Authors:** André Dias-Frias, Ricardo Costa, Andreia Campinas, André Alexandre, David Sá-Couto, Maria João Sousa, Carla Roque, Pinheiro Vieira, Vitor Lagarto, Hipólito Reis, Severo Torres

**Affiliations:** Centro Hospitalar Universitário do Porto, 4099-001 Porto, Portugal

**Keywords:** cardiac pacing, heart failure, mortality, right ventricular septal pacing, right ventricular apical pacing

## Abstract

The clinical benefits of right ventricular septal (RVS) pacing compared to those of right ventricular apical (RVA) pacing are still in debate. We aimed to compare the incidence of heart failure (HF) and all-cause mortality in patients submitted to RVS and RVA pacing during a longer follow-up. This a single-center, retrospective study analysis of consecutive patients submitted to pacemaker implantation. The primary outcome was defined as the occurrence of HF during follow-up. The secondary outcome was all-cause death. A total of 251 patients were included, 47 (18.7%) with RVS pacing. RVS pacing was associated to younger age, male gender, lower body mass index, ischemic heart disease, and atrial fibrillation. During a follow-up period of 5.2 years, the primary outcome occurred in 89 (37.1%) patients. RVS pacing was independently associated with a 3-fold lower risk of HF, after adjustment. The secondary outcome occurred in 83 (34.2%) patients, and pacemaker lead position was not a predictor. Fluoroscopy time and rate of complications (rarely life-threatening) were similar in both groups. Our study points to a potential clinical benefit of RVS positioning, with a 3.3-fold lower risk of HF, without accompanying increase in procedure complexity nor complication rate.

## 1. Introduction

Cardiac pacemaker remains the most effective treatment of several bradyarrhythmias. Worldwide, one million patients are submitted to pacemaker implantation every year and this number is increasing [1]; however, chronic right ventricular (RV) pacing may be deleterious, leading to pacing-induced cardiomyopathy in 10–20% of patients after 2–4 years [2,3,4]. 

There is lack of consensus on the optimal positioning of the RV pacing lead. Conventionally, RV lead is placed at the apex, but alternative locations have been studied; one of these is pacing of the RV septum. The rationale is that septal pacing might recruit part of the intrinsic cardiac conduction system that lies in close proximity, thereby reducing QRS duration and subsequent ventricular dyssynchrony [5,6,7,8,9,10]. 

Many previous studies comparing RV septal (RVS) and RV apical (RVA) pacing did not show significantly different clinical outcomes [11,12,13]; however, most of them had a relatively short follow-up period.

In this study, we aimed to compare the long-term incidence of heart failure (HF) and all-cause mortality in patients submitted to RVS and RVA pacing during a longer follow-up period than in previous studies. 

## 2. Materials and Methods

We conducted a retrospective single-center study of consecutive patients submitted to pacemaker implantation with RV pacing lead at a tertiary academic hospital during 2015. Pacemaker implantation was performed according to the 2013 ESC Guidelines on cardiac pacing and cardiac resynchronization therapy recommendations [14]. RV lead location was chosen by the pacemaker implantation operator and confirmed by fluoroscopic projections.

All data of demographic, clinical, laboratory, echocardiographic, and pacemaker parameters were collected by review of electronic health records.

All baseline clinical characteristics were collected from index pacemaker implantation hospitalization. Left ventricular ejection fraction (LVEF) was calculated by echocardiographic biplane Simpson’s method, according to the European Association of Cardiovascular Imaging recommendations [15]. Chronic kidney disease was defined as an estimated glomerular filtration rate < 60 mL/min/1.37 m^2^ by CKD-EPI Creatinine formula [16].

The primary outcome was defined as the occurrence of HF during follow-up. HF was defined either by a (1) HF hospitalization or (2) the worsening of the patient’s symptoms or signs of congestion that led to de novo prescription or up-titration of diuretics by the assistant physician in the outpatient clinic. The secondary outcome was defined by the occurrence of all-cause death during follow-up. They were all assessed by electronic health record review.

Statistical analysis was conducted using IBM SPSS Statistics for Windows (version 24.0. Armonk, NY, USA: IBM Corp.). Independent student’s t-test and Mann–Whitney U test were used to compare normally and non-normally distributed continuous variables, respectively. Categorical variables were compared by chi-square or Fisher’s exact tests. Independent predictors of the outcomes were assessed using Cox models. We adjusted the Cox proportional hazard models for the following variables: age, sex, comorbidities such as hypertension, dyslipidemia, diabetes mellitus, smoking, ischemic heart disease, atrial fibrillation, previous diagnosed HF, peripheral arterial disease and chronic kidney disease, serum levels of hemoglobin and creatinine at hospital admission, and baseline echocardiographic findings as LVEF, right ventricular systolic function, and severe valvular heart disease. The Kaplan–Meier method was used to estimate the survival curves. All tests were two-sided and *p*-value < 0.05 was considered to be statistically significant.

The study was approved by the local ethics committee and conducted in accordance with the Declaration of Helsinki.

## 3. Results

During the study period, 251 patients were submitted to pacemaker implantation, 47 (18.7%) with RVS pacing and 204 (81.3%) with RVA pacing. During a median follow-up period of 5.2 (IQR 3.0–5.5) years, the primary outcome occurred in 89 (37.1%) patients and the secondary outcome in 83 (34.2%) patients.

Baseline characteristics of the overall population are displayed in Table 1. The mean age was 76.5 ± 11.3 years and 129 (51.4%) were male. RVS pacing was significantly associated to younger age, male gender, lower body mass index, ischemic heart disease, and atrial fibrillation. Patients submitted to RVS pacing were also less frequently medicated with antiplatelet agents. At hospital admission, they had higher values of hemoglobin. More than one third (36.7%) of the patients had previously diagnosed HF. Mean LVEF before pacemaker implantation was 54.1 ± 7.5%, and was similar between groups. 

The main indication to pacemaker implantation was atrioventricular block (69.3%) and it was even more frequent in the RVS pacing group (85.1%). A lower proportion of patients in this group received a dual-chamber pacemaker than the RVA pacing group (53.2% vs. 71.1%, *p* = 0.02). Mean QRS duration before pacemaker implantation was similar between groups (116.0 ± 25.8 milliseconds). Median radiation time during procedure was 3.2 (interquartile range (IQR) 1.6–5.5) minutes, with no significant difference between groups.

Seven (2.8%) patients died during hospitalization, none related to pacemaker implantation complications.

**Figure 1:**
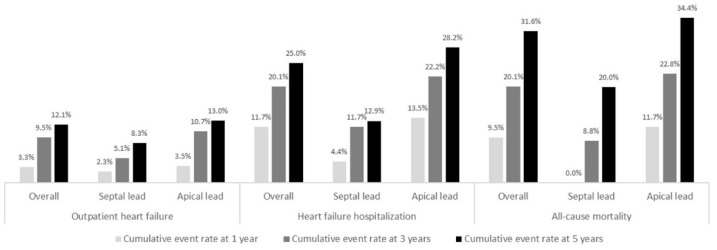
Cumulative heart failure and all-cause mortality event rate at 1, 3, and 5 years of follow-up.

**Figure 2:**
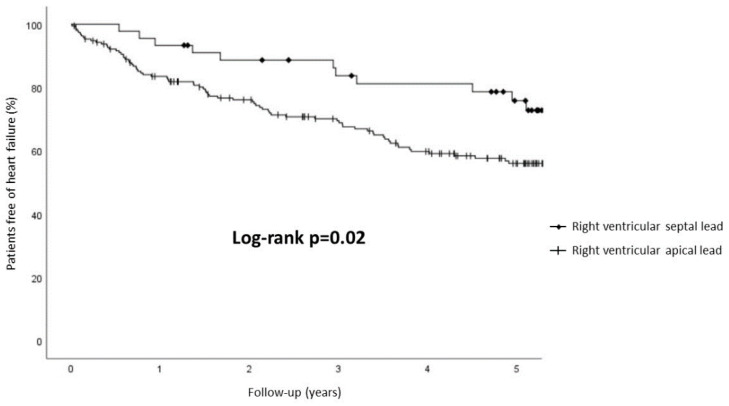
Heart failure during follow-up in patients with septal and apical right ventricular pacing lead.

Follow-up variables of the overall population are displayed in Table 2. During a median follow-up period of 5.2 (IQR 3.0–5.5) years, the primary outcome occurred in 89 (37.1%) patients; 29 (12.1%) managed as outpatient and 60 (25.0%) requiring hospitalization (Figure 1). Median time-to-event was 1.5 (IQR 0.6–3.3) years. Cumulative event rate at 1 and 3 was 15.0% and 29.6%, respectively (Figure 1). RVS pacing was significantly associated to a lower incidence of HF (Figure 2). In multivariate analysis (Table 3), RVA pacing, previous diagnosis of HF, and severe aortic valvular stenosis were independent predictors of the primary outcome, but not the percentage of RV pacing.

During the follow-up period, 83 (34.2%) patients died. Median time-to-event was 2.6 (IQR 0.9–4.2) years. Cumulative event rate at 1 and 3 years was 9.5% and 20.1%, respectively (Figure 2). Independent predictor (Supplementary Table S1) was solely age.

Median percentage of ventricular pacing was 84% (IQR 16–99), 67% of them with ≥40%, without significant differences between groups. QRS duration during RV pacing was available in 105 (43.0%) patients and it was lower in RVS pacing group. LVEF during follow-up was available in 135 (55.3%) subjects and it was lower than 50% in 35.6% of patients, and similar between groups. Upgrade to cardiac resynchronization therapy was performed in 2.5% of patients (three subjects in each group); however, in each group, only one of these had LVEF ≥ 50% at baseline.

Regarding the complications, there was one subclavian vein thrombosis in the RVA pacing group, one diaphragm stimulation requiring RV lead repositioning, and one pacemaker lead-related infective endocarditis in the RVS pacing group, and one pocket infection and one significant pocket hematoma in each group.

## 4. Discussion

In this contemporary cohort of consecutive patients submitted to pacemaker implantation, 37% of them developed HF during a median follow-up period of 5 years. RVS pacing was independently associated to a 3.3-fold lower risk compared to RVA pacing. The median interval between pacemaker implantation and incident HF was approximately 1.5 years. All-cause mortality incidence was 34%. The number of complications was low and non-life-threatening. RVS pacing was not associated to higher radiation time during implantation procedure. As far as we know, our study is the first showing a lower incidence of HF in patients with RVS pacing compared to those submitted to the gold standard RVA pacing.

In previous studies, RVS pacing was associated to less ventricular dyssynchrony but there were no differences regarding clinical and echocardiographic outcomes [5,6,7,8,9,10,11,12,13,17,18]; in fact, due to the lack of evidence, the recent 2021 ESC Guidelines on cardiac pacing and cardiac resynchronization therapy do not state any recommendation preferring one RV pacing site over the other [19]. Considering previous studies that evaluated HF and all-cause mortality as outcome, some differences in the population and the study design could explain our divergent results [11,12,13]. First, they had a much shorter follow-up period (less than 3 years), although in our study we saw differences between RVS and RVA pacing groups early in the first year of follow-up. Another important feature is the prevalence of comorbidities typically associated to HF events and death, as atrial fibrillation, previous diagnosis of HF, and LVEF < 50% before pacemaker implantation that were higher in our cohort. In fact, the incidence of these outcomes was also higher: 37% versus 3–9% of incident HF and 34% versus 7–29% of all-cause death. In contrast with previous data that only considered HF hospitalizations, we also included outpatient HF event as a clinically relevant outcome. It was recently shown that these episodes are associated with a 3-fold higher risk of death in comparison with patients without outpatient HF worsening [20]. Finally, in our cohort, patients submitted to RVS pacing had a significantly narrower QRS during RVS pacing compared to the RVA pacing group, as previously reported in some, but not all, studies [21,22].

Well-known risk factors of HF such as atrial fibrillation and ischemic heart disease were more frequent in patients submitted to RVS pacing, which reinforces our results; also, these patients were younger and there was a tendency to be associated to LVEF < 50% before pacemaker implantation, which could reflect the belief of the assistant physician that this strategy could lead to a better outcome. We thought that the last could be the cause of the higher number of RVS pacing patients being submitted to upgrade to cardiac resynchronization therapy; however, despite the adjusted analysis, we should not ignore the fact that older patients, with lower hemoglobin and other comorbidities that could not be captured in our study, may have led to a worse outcome than those in the RVA pacing group. 

Regarding all-cause mortality, it was lower in RVS pacing patients but this effect was not observed in the adjusted analysis. One previous retrospective study showed a mortality benefit of RVS pacing on a 5-year follow-up [23]. We suspect this difference is mainly driven by a smaller sample in our study.

RVS pacing typically raises concerns about the increase in technical complexity with longer procedures and the potential risk of RV wall perforation and lead displacement; nonetheless, both techniques were associated to a low number of complications, and the most serious was one non-fatal case of device-related endocarditis in the RVS pacing group; also, fluoroscopy time was not different in the two groups. 

Our study has limitations that should be considered: firstly, it was not a randomized controlled trial and, due to that, there were important clinical differences between the two groups; secondly, its retrospective design may lead to a potential bias due to dependency on the accuracy of clinical records; thirdly, it was a single-center study and the extrapolation of our results should be cautious; and, finally, data about the exact position of the RV septal pacing lead site was not available.

In order to reduce the burden of pacing-induced cardiomyopathy and consequent morbimortality, randomized controlled trials with longer follow-up periods are needed to confirm our findings, especially considering that RVS pacing is already performed in several centers worldwide and it is less technically challenging than other strategies, such as cardiac-resynchronization therapy and His-bundle pacing.

## 5. Conclusions

Our study shows that RVS pacing is independently associated to a 3.3-fold lower risk of incident HF compared to RVA pacing, without a significant increase in procedure complexity nor higher complication rate. Randomized control trials with long follow-up are needed to confirm our findings.

## Figures and Tables

**Table 1:** Baseline characteristics of studied population, by the location of the right ventricular pacing lead.

Baseline Characteristics	Overall (n = 251)	Septal (n = 47)	Apical (n = 204)	*p* Value
Age (years), mean (SD)	76.5 (11.3)	72.2 (11.4)	77.4 (11.1)	0.004
Men	129 (51.4%)	32 (68.1%)	97 (47.5%)	0.008
BMI (Kg/m^2^), mean (SD)	28.0 (5.2)	26.6 (4.3)	28.4 (5.4)	0.01
Hypertension	190 (75.7%)	31 (66.0%)	159 (77.9%)	0.07
Dyslipidemia	131 (52.2%)	27 (57.4%)	104 (51.0%)	0.26
Diabetes mellitus	80 (32.0%)	16 (34.0%)	64 (31.5%)	0.43
Insulin-treated	23 (9.3%)	2 (4.5%)	21 (10.3%)	0.23
Smoker	53 (21.1%)	11 (23.4%)	42 (20.6%)	0.40
Coronary artery disease	48 (19.1%)	14 (29.8%)	34 (16.7%)	0.04
Heart failure	92 (36.7%)	15 (31.9%)	77 (37.7%)	0.28
Atrial fibrillation	101 (40.2%)	26 (55.3%)	75 (36.8%)	0.02
Peripheral artery disease	9 (3.6%)	2 (4.3%)	7 (3.4%)	0.78
Chronic kidney disease	66 (26.3%)	9 (19.1%)	57 (27.9%)	0.15
Hemoglobin (g/dL), mean (SD)	12.9 (1.8)	13.5 (1.8)	12.7 (1.7)	0.01
Serum creatinine, median (IQR)	0.98 (0.80–1.34)	0.90 (0.78–1.22)	1.00 (0.80–1.40)	0.14
Medication prior to pacemaker implantation				
Antiplatelet	89 (36.0)	10 (22.7%)	79 (38.9%)	0.03
Vitamin K antagonist	49 (19.8)	13 (29.5%)	36 (17.7%)	0.06
Novel oral anticoagulant	27 (10.9)	8 (18.2%)	19 (9.4%)	0.09
Beta-blocker	59 (23.8)	9 (20.5%)	50 (24.5%)	0.36
Ivabradine	1 (0.4)	1 (2.3%)	0	0.18
Cardiac glycoside	7 (2.8%)	2 (4.5%)	5 (2.5%)	0.45
ACE-I/ARB	142 (57.3%)	21 (47.7%)	121 (59.3%)	0.38
MRA	10 (4.0%)	0	10 (4.9%)	0.13
Loop diuretic	88 (35.5%)	12 (27.3%)	76 (37.3%)	0.14
LVEF (%), mean (SD)	54.1 (7.5)	53.7 (7.5)	54.2 (7.6)	0.71
LVEF < 50%	36 (17.0%)	10 (24.4%)	26 (15.2%)	0.12
RV systolic dysfunction	12 (5.7%)	2 (5.0%)	10 (5.8%)	0.84
Severe LVH	6 (2.8%)	0	6 (3.5%)	0.23
Large pericardial effusion	2 (1.0%)	0	2 (1.2%)	0.49
Severe AS	9 (4.3%)	2 (5.0%)	7 (4.1%)	0.80
Significant MS	2 (0.9%)	0	2 (1.2%)	0.49
Severe MR	1 (0.5%)	0	1 (0.6%)	0.63
Severe TR	3 (1.4%)	1 (2.5%)	2 (1.2%)	0.52
Pacemaker indication				
Sinus node disease	38 (15.1%)	3 (6.4%)	35 (17.2%)	0.04
Atrioventricular block	174 (69.3%)	40 (85.1%)	134 (65.7%)	0.006
Tachycardia-bradycardia syndrome	39 (15.5%)	4 (8.5%)	35 (17.2%)	0.10
QRS duration (milliseconds), mean (SD)	116.0 (25.8)	112.5 (22.6)	116.6 (26.4)	0.36
Dual-chamber pacemaker	170 (67.7%)	25 (53.2%)	145 (71.1%)	0.02
Radiation time, median (IQR)	3.2 (1.6–5.5)	3.3 (2.3–5.5)	3.1 (1.6–5.5)	0.53
In-hospital mortality	7 (2.8%)	1 (2.1%)	6 (2.9%)	0.76

ACE-I: angiotensin-converting-enzyme inhibitor; ARB: angiotensin II receptor blocker; ARNI: angiotensin-receptor neprilysin inhibitor; AS: aortic stenosis; BMI: body mass index; IQR: interquartile range; LVEF: left ventricular ejection fraction; LVH: left ventricular hypertrophy; MR: mitral regurgitation; MRA: mineralocorticoid receptor antagonist; MS: mitral stenosis; SD: standard deviation; TR: tricuspid regurgitation.

**Table 2:** Follow-up characteristics of studied population, by the location of the right ventricular pacing lead.

Follow-Up	Overall (n = 244)	Septal (n = 46)	Apical (n = 198)	*p* Value
Follow-up time, median (IQR)	5.2 (3.0–5.5)	5.3 (5.1–5.5)	5.1 (2.6–5.4)	0.01
QRS duration during RV pacing (milliseconds), mean (SD)	171.1 (21.1)	159.0 (13.3)	173.9 (21.6)	<0.001
Pacemaker complications				
Pocket infection	2 (0.8%)	1 (2.1%)	1 (0.5%)	0.26
Pocket hematoma	2 (0.8%)	1 (2.1%)	1 (0.5%)	0.26
Subclavian vein thrombosis	1 (0.4%)	0	1 (0.5%)	0.63
Diaphragm stimulation requiring lead repositioning	1 (0.4%)	1 (2.1%)	0	0.19
Pacemaker related endocarditis	1 (0.4%)	1 (2.1%)	0	0.19
Heart failure	89 (37.1%)	11 (23.9%)	78 (40.2%)	0.03
Outpatient	29 (12.1%)	4 (8.7%)	25 (12.9%)	0.31
Hospitalization	60 (25.0%)	7 (15.2%)	53 (27.3%)	0.06
Time-to-heart failure, median (IQR)	1.5 (0.6–3.3)	2.9 (0.9–4.5)	1.5 (0.6–3.2)	0.16
All-cause mortality	83 (34.2%)	9 (19.6%)	74 (37.6%)	0.01
Time-to-death, median (IQR)	2.6 (0.9–4.2)	3.1 (2.0–4.4)	2.3 (0.8–4.3)	0.31
RV pacing percentage	84 (16–99)	85 (18–98)	84 (16–99)	0.99
RV pacing percentage ≥ 40%	139 (66.5%)	29 (70.7%)	110 (65.5%)	0.33
LVEF (%), mean (SD)	50.9 (9.9)	50.1 (9.2)	51.2 (10.2)	0.56
LVEF < 50%	48 (35.6%)	15 (45.5%)	33 (32.4%)	0.12
Upgrade to CRT	6 (2.5%)	3 (6.5%)	3 (1.5%)	0.05
Medication during follow-up				
Antiplatelet	76 (32.3%)	11 (25.0%)	65 (34.0%)	0.17
Vitamin K antagonist	41 (17.4%)	13 (29.5%)	28 (14.7%)	0.02
Novel oral anticoagulant	55 (23.4%)	14 (31.8%)	41 (21.5%)	0.11
Beta-blocker	96 (40.9%)	19 (43.2%)	77 (40.3%)	0.43
Ivabradine	4 (1.7%)	2 (4.5%)	2 (1.0%)	0.11
Cardiac glycoside	18 (7.7%)	3 (6.8%)	15 (7.9%)	0.82
ACE-I/ARB	113 (48.3%)	25 (56.8%)	88 (46.3%)	0.14
ARNI	5 (2.1%)	2 (4.5%)	3 (1.6%)	0.22
MRA	17 (7.2%)	4 (9.1%)	13 (6.8%)	0.60
Loop diuretic	120 (51.0%)	19 (43.2%)	101 (52.9%)	0.16
SGLT2 inhibitor	10 (4.3%)	4 (9.1%)	6 (3.1%)	0.08
GLP-1 receptor agonist	2 (0.9%)	1 (2.3%)	1 (0.5%)	0.26

ACE-I: angiotensin-converting-enzyme inhibitor; ARB: angiotensin II receptor blocker; ARNI: angiotensin-receptor neprilysin inhibitor; CRT: cardiac resynchronization therapy; GLP-1: glucagon-like peptide-1; IQR: interquartile range; LVEF: left ventricular ejection fraction; MRA: mineralocorticoid receptor antagonist; RV: right ventricle; SD: standard deviation; SGLT2: sodium-glucose co-transporter-2.

**Table 3:** Univariate and multivariate analysis of predictors of the primary outcome.

Variable	Univariate Analysis		Multivariate Analysis	
	HR 95% CI	*p* Value	HR 95% CI	*p* Value
Age (years)	1.04 (1.01–1.06)	0.002	1.01 (0.99–1.04)	0.46
Men	1.25 (0.82–1.89)	0.30	1.59 (0.95–2.65)	0.08
Hypertension	1.53 (0.89–2.63)	0.12	-	-
Dyslipidemia	1.65 (1.07–2.55)	0.02	-	-
Diabetes mellitus	1.57 (1.03–2.40)	0.04	-	-
Smoker	1.62 (1.03–2.56)	0.04	-	-
Coronary artery disease	1.97 (1.23–3.15)	0.005	1.66 (0.95–2.91)	0.08
Heart failure	3.84 (2.50–5.89)	<0.001	3.22 (1.88–5.5)	<0.001
Atrial fibrillation	1.60 (1.05–2.42)	0.03	-	-
Chronic kidney disease	1.97 (1.25–3.09)	0.003	-	-
Hemoglobin (g/dL)	0.85 (0.76–0.96)	0.008	-	-
Serum creatinine (mg/dL)	1.13 (0.97–1.31)	0.11	-	-
LVEF < 50%	2.55 (1.55–4.22)	<0.001	1.46 (0.76–2.79)	0.26
RV systolic disfunction	1.26 (0.46–3.46)	0.65	-	-
Severe AS	6.20 (2.66–14.42)	<0.001	10.88 (3.25–36.44)	<0.001
Significant MS	1.71 (0.24–12.35)	0.60	-	-
Severe MR	4.35 (0.60–31.64)	0.15	-	-
Severe TR	1.68 (0.41–6.86)	0.47	-	-
RVA pacing	2.08 (1.11–4.00)	0.02	3.32 (1.48–7.46)	0.004
RV pacing percentage	1.00 (0.99–1.01)	0.90	1.00 (0.99–1.01)	0.74

AS: aortic stenosis; CI: confidence interval; HR: hazard ratio; LVEF: left ventricular ejection fraction; MR: mitral regurgitation; MS: mitral stenosis; RV: right ventricular; RVA: right ventricular apical; SD: standard deviation; TR: tricuspid regurgitation.

## Data Availability

The data presented in this study are available on request from the corresponding author. The data are not publicly available due to patient confidentiality.

## Supplementary Materials

The following supporting information can be downloaded at: https://www.mdpi.com/article/10.3390/jcdd9120444/s1, Table S1: Univariate and multivariate analysis of predictors of the secondary outcome.
