# Supplementary material for: Right Ventricular Septal Versus Apical Pacing: Long-Term Incidence of Heart Failure and Survival
Source: J Cardiovasc Dev Dis. 2022 Dec 9;9(12):444. doi: 10.3390/jcdd9120444 (PMC9786931; doi:10.3390/jcdd9120444)
Supplement: Supplementary file 1 [file jcdd-09-00444-s001.zip › jcdd-2003756-supplementary.pdf]

Table S1. Univariate and multivariate analysis of predictors of the secondary outcome.

Variable	Univariate Analysis		Multivariate Analysis	
	HR 95% CI	*p* Value	HR 95% CI	*p* Value
Age (years)	1.11 (1.08–1.15)	<0.001	1.11 (1.06–1.15)	<0.001
Men	0.90 (0.58–1.38)	0.62	1.40 (0.74–2.62)	0.30
Hypertension	1.50 (0.86–2.62)	0.16	-	-
Dyslipidemia	0.72 (0.47–1.11)	0.13	-	-
Diabetes mellitus	1.39 (0.89–2.16)	0.15	-	-
Smoker	0.84 (0.49–1.44)	0.53	-	-
Coronary artery disease	1.40 (0.84–2.33)	0.20	-	-
Heart failure	2.38 (1.55–3.67)	<0.001	1.03 (0.52–2.04)	0.94
Atrial fibrillation	1.09 (0.70–1.68)	0.71	-	-
Chronic kidney disease	2.34 (1.50–3.65)	<0.001	1.29 (0.63–2.62)	0.49
Hemoglobin (g/dL)	0.79 (0.70–0.89)	<0.001	-	-
Serum creatinine (mg/dL)	1.18 (1.02–1.38)	0.03	-	-
LVEF <50%	1.74 (0.98–3.09)	0.06	1.45 (0.47–4.49)	0.58
RV systolic disfunction	2.66 (1.15–6.16)	0.02	2.97 (0.51–17.14)	0.22
Severe AS	2.80 (0.87–8.98)	0.08	-	-
Significant MS	1.60 (0.22–11.54)	0.64	-	-
Severe MR	1.47 (0.81–2.79)	0.65	-	-
Severe TR	1.01 (0.14–7.30)	0.99	-	-
RVA pacing	2.33 (1.16–4.55)	0.02	1.74 (0.71–4.24)	1.74
RV pacing percentage	1.01 (1.00–1.01)	0.15	1.00 (0.99–1.01)	0.46

AS: aortic stenosis; CI: confidence interval; HR: hazard ratio; LVEF: left ventricular ejection fraction; MR: mitral regurgitation; MS: mitral stenosis; RV: right ventricular; RVA: right ventricular apical; SD: standard deviation; TR: tricuspid regurgitation.
